# Copper ferrites@reduced graphene oxide anode materials for advanced lithium storage applications

**DOI:** 10.1038/s41598-017-09214-0

**Published:** 2017-08-21

**Authors:** Junyong Wang, Qinglin Deng, Mengjiao Li, Kai Jiang, Jinzhong Zhang, Zhigao Hu, Junhao Chu

**Affiliations:** Key Laboratory of Polar Materials and Devices (MOE) and Technical Center for Multifunctional Magneto-Optical Spectroscopy (Shanghai), Department of Electronic Engineering, East China Normal University, Shanghai, 200241 China

## Abstract

Copper ferrites are emerging transition metal oxides that have potential applications in energy storage devices. However, it still lacks in-depth designing of copper ferrites based anode architectures with enhanced electroactivity for lithium-ion batteries. Here, we report a facile synthesis technology of copper ferrites anchored on reduced graphene oxide (CuFeO_2_@rGO and Cu/CuFe_2_O_4_@rGO) as the high-performance electrodes. In the resulting configuration, reduced graphene offers continuous conductive channels for electron/ion transfer and high specific surface area to accommodate the volume expansion of copper ferrites. Consequently, the sheet-on-sheet CuFeO_2_@rGO electrode exhibits a high reversible capacity (587 mAh g^−1^ after 100 cycles at 200 mA g^−1^). In particular, Cu/CuFe_2_O_4_@rGO hybrid, which combines the advantages of nano-copper and reduced graphene, manifests a significant enhancement in lithium storage properties. It reveals superior rate capability (723 mAh g^−1^ at 800 mA g^−1^; 560 mAh g^−1^ at 3200 mA g^−1^) and robust cycling capability (1102 mAh g^−1^ after 250 cycles at 800 mA g^−1^). This unique structure design provides a strategy for the development of multivalent metal oxides in lithium storage device applications.

## Introduction

Rechargeable lithium-ion batteries (LIBs) with high energy density and power density have been widely used as energy storage devices^1–5^. There is an increasing demand for LIBs with long-term stability, safety and low cost to meet future requirements for consumer electronics and electric vehicles^6, 7^. Therefore, searching for new anode materials with ultrahigh theoretical capacity and remarkable electrochemical performance is urgently required^8, 9^, due to the low theoretical capacity for the current commercial graphite anodes^10^. Considerable researches have been devoted to the design of transition metal oxides (TMOs) based electrodes including the binary, ternary, and complex metal oxides, for application in high-performance energy storage devices^11–18^. Among TMOs, the ternary oxides with delafossite structure (ABO_2_) and spinel structure (AB_2_O_4_) have unique layered crystal structures with three-dimensional diffusion pathways, which are benefit for lithium ion insertion and extraction^19–23^. Recently, CuCo_2_O_4_ and ZnFe_2_O_4_ have been investigated as anode materials, which exhibit good reversible capacity and cyclability and guide the following study on the ternary oxide anode materials^24, 25^.

Compared with them, copper ferrites including CuFeO_2_ and CuFe_2_O_4_ have been considered as promising anode materials for the advantages of natural abundance, environmental friendliness, high specific and practical availability^26–30^. The CuFeO_2_ anode materials for LIBs was first reported by Lu’s group in 2011^31^. CuFeO_2_ and graphene composites, which had a specific capacity of 670 mAh g^−1^, were prepared by a low temperature hydrothermal method^32^. As the anode for LIBs, pure CuFe_2_O_4_ with different morphologies have been investigated^33–36^. Carbon coated hollow CuFe_2_O_4_ spheres with specific capacity of 550 mAh g^−1^ was obtained by a polymer-template hydrothermal growth method^37^. Polypyrrole-coated CuFe_2_O_4_ for LIBs with enhanced electrochemical performance was reported by the electrostatic spray deposition technique^38^. Unfortunately, the application of copper ferrites in LIBs have been impeded by the inherent sluggish kinetic and large volume expansion/contraction during cycling, which eventually leads to rapid capacity fading and poor cycling stability. Some strategies have been achieved to overcome these obstacles, such as downsizing crystal size^39^, designing various porous structures^32^, hierarchical structures^40, 41^.

Recently, the development of nanotechnology provides more approaches to manufacture optimized architecture for enhancing the electrochemical active of copper ferrites. The availability of small crystal size with high specific surface area and facile stress relaxation processes effectively facilitates the Li^+^ diffusion and makes high rate capability possible^42–44^. Furthermore, graphene with high surface area and great mechanical stiffness have been widely used for energy storage devices as a conductive additive to enhance the electrochemical reactions^45–47^. In particular, few-layered graphene (FLG) obtained from graphene oxide exhibits a high reversible capacity and excellent Columbic efficiency and very low cycle to cycle capacity fading^48–51^. The addition of graphene can not only increase electrode-electrolyte contact area and faster electrolyte access to active materials, but also mitigate the volume change and limit structure degradation during cycling^52, 53^. Moreover, the integration of metallic nanocrystals is a new strategy to address the weak charge transfer kinetics for high active surface/interface and robust stability^40, 54, 55^. Therefore, the design of hybrid architectures with improved capability and stability are highly necessary to achieve prominent performances for copper ferrites anodes.

**Figure 1:**
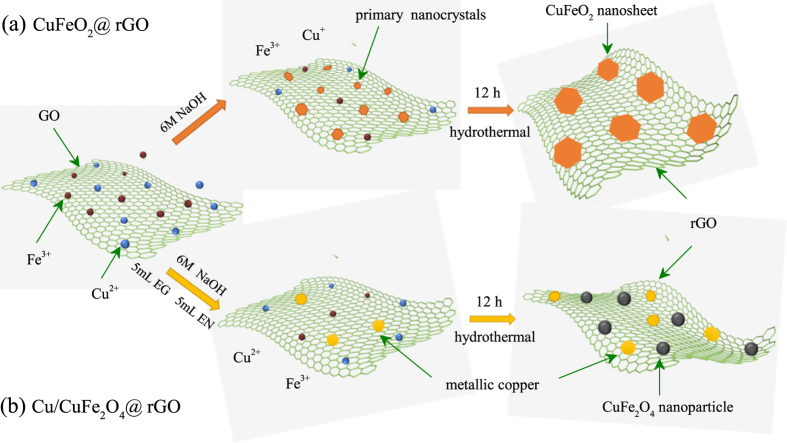
Schematic illustration of the synthesis of (**a**) CuFeO_2_@rGO and (**b**) Cu/CuFe_2_O_4_@rGO.

Herein, we report an efficient and scalable hydrothermal method for synthesizing the copper ferrites@rGO composites (CuFeO_2_@rGO and Cu/CuFe_2_O_4_@rGO) with boosted electrochemical performance. As illustrated in Fig. 1, the copper and iron ions could adsorb on the exposed GO surface owing to the oxygen-containing groups after being well dispersed in GO suspension. In the fabrication processes of CuFeO_2_@rGO composites (Fig. 1a), the primary delafossite CuFeO_2_ nanocrystals are first formed in the NaOH solution, followed by the oriented attachment growth to construct the sheet-on-sheet CuFeO_2_@rGO architecture. The synergistic effects of each component improve the capability of CuFeO_2_@rGO electrode (587 mAh g^−1^ at 200 mA g^−1^ after 100 cycles). As shown in Fig. 1b, the Cu/CuFe_2_O_4_@rGO composite was obtained by means of the reducing and complex characteristics of ethylene glycol (EG) and the stronger complexing ability of ethylenediamine (EN). In this reaction system, part of copper ions form CuFe-precursor combining with iron ions. The rest of copper ions were reduced to metallic copper through reacting with ethylene glycol and ethanediamine in the liquid solution. The similar mechanism for preparing spinel compound oxides by using non-stoichiometric ratio have been reported^40, 41^. Such a phase transformation leads to the well distribution of CuFe_2_O_4_ and copper on the surface of rGO, while each of them is interconnected by graphene. It is anticipated that such Cu/CuFe_2_O_4_@rGO configuration can gain access to the following advantages: (i) a sufficient electrical contact for rapid electron transfer and a shorter channel for fast lithium ion transport, promoting the electrode reaction kinetic; (ii) large surface area ensures effective contact between the electrolyte and electrode, enhancing the electrochemical actively; (iii) available internal voids can buffer the volume change during lithiation/delithiation processes, increasing structural stability. Unsurprisingly, the as-built Cu/CuFe_2_O_4_@rGO electrode exhibited a remarkable rate capability (560 mAh g^−1^ at 3200 mA g^−1^) and cycling stability (835.2 mAh g^−1^ over 100 cycles at 200 mA g^−1^), indicating a promising prospect of application in high-end energy storage devices.

## Results and Discussion

### The CuFeO_2_@rGO composites

**Figure 2:**
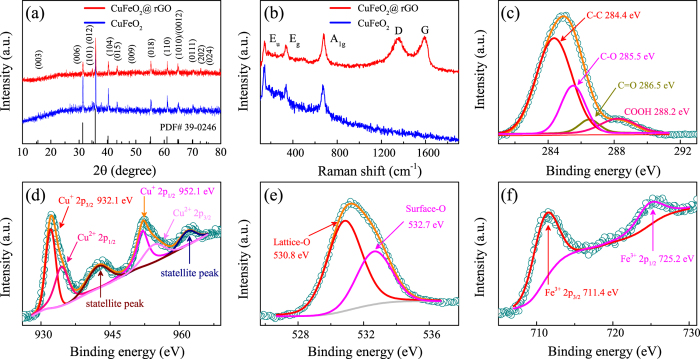
(**a**) XRD patterns and (**b**) Raman spectra of CuFeO_2_ and CuFeO_2_@rGO. High-resolution XPS spectra of (**c**) C 1 *s*, (**d**) Cu 2*p*, (**e**) O 1 *s*, and (**f**) Fe 2*p* for CuFeO_2_@rGO.

The crystalline phase and composition of as-synthesized CuFeO_2_@rGO composites were investigated by XRD measurements. As shown in Fig. 2a, the single delafossite CuFeO_2_ phase (PDF# 39-0246) of CuFeO_2_@rGO can be well observed in the scan range of 10–80°, which confirms the good crystallinity of the samples. As for the pure CuFeO_2_, CuFeO_2_ is the predominating phase along with a weak impure peak located at around 38°, which may be related to CuO or 2H-CuFeO_2_. It is hard to determined due to its weak intensity. Pure CuFeO_2_ displays sharper and stronger diffraction peaks than CuFeO_2_@rGO, suggesting the smaller crystallite size in CuFeO_2_@rGO. The d-spacings of the (006) and (110) diffraction peaks indicate that lattice parameters of a = 0.3031 nm and c = 1.7141 nm for CuFeO_2_@rGO sample. In comparison, pure CuFeO_2_ has lattice parameter values of a = 0.3027 nm and c = 1.7161 nm. Raman spectroscopy was adopted to evaluate the graphitic quality, which cannot be detected by XRD data (Fig. 2b). There are three modes at around 105, 342 and 670 cm^−1^, which correspond to E_*u*_, E_*g*_ and A_1*g*_ of delafossite CuFeO_2_^56^. In addition, two well-resolved bands at 1360 and 1590 cm^−1^ for CuFeO_2_@rGO are attributed to the D band (k-point phonon of A_1*g*_ symmetry) and G band (E_2*g*_ phonon of carbon) of graphene, respectively. Compared with GO (*I*_*D*_/*I*_*G*_ = 0.86), the increased ratio of the D band to G band (*I*_*D*_/*I*_*G*_ = 0.97) in CuFeO_2_@rGO suggests the reduction of graphene, which can be ascribed to smaller but more numerous sp^2^ domains in carbon^57^. Moreover, the presence of 2D band at 2694 cm^−1^ and (D + G) band at 2953 cm^−1^ in Fig. S1 manifests a substantial increase in the disorder degree in graphene sheets. The high ratio *I*_*G*_/*I*_2*D*_ indicates the relatively thick graphene layers^48, 50^. The specific surface area of as-prepared composites was calculated by the Brunauer-Emmett-Teller (BET) method. As shown in Fig. S2, the BET surface area of CuFeO_2_@rGO is 14.63 m^2^ g^−1^, which is very low specific surface area in comparison with graphene^51^. It indicates that lots of the active surface of graphene have been covered by CuFeO_2_ crystal with smaller specific surface area^16^, which may not readily allow nitrogen molecules to get adsorbed onto them unlike pristine graphene. XPS measurements were conducted to detect the composition and chemical state of CuFeO_2_@rGO. The full XPS spectrum (Fig. S3) reveals the presence of Cu 2p, Fe 2p, O 1 s and C 1 s, with no evidence of impurities. Figure 2d shows the high resolution XPS spectrum of Cu 2p. The dominant doublet peaks positioned at 932.1 and 952.1 eV are ascribed to Cu 2p_3/2_ and Cu 2p_1/2_ for Cu^+^ from CuFeO_2_^58^. The shoulder peaks located at around 934.5 and 954.9 eV along with two satellite peaks at 943.1 and 961.9 eV correspond to Cu 2p_3/2_ and Cu 2p_1/2_ for Cu^2+^, which should be caused by the easy oxidation of Cu in air atmosphere^29^. The O 1 s peak located at 530.8 eV (Fig. 2e) further confirms the formation of CuFeO_2_^59^, whereas the higher binding energy peak positioned at about 532.7 eV is attributed to the surface adsorbed hydroxyl oxygen^21, 60^. The high resolution XPS spectrum of the Fe 2p doublet (Fig. 2f) with two peaks located at 711.4 eV for Fe 2p_3/2_ and 725.2 eV for Fe 2p_1/2_, is characteristic of Fe^3+^ ^60^. In addition, the high resolution C 1 s spectrum in Fig. 2c could be deconvoluted into four peaks at 284.4, 285.4, 286.5 and 288.2 eV, corresponding to C-C, C-O, C=O and COOH bonds, respectively. Note that the C-C bond is dominated in the C functional groups, which indicates the possibility of electronic conductivity improvement for CuFeO_2_@rGO electrode.

**Figure 3:**
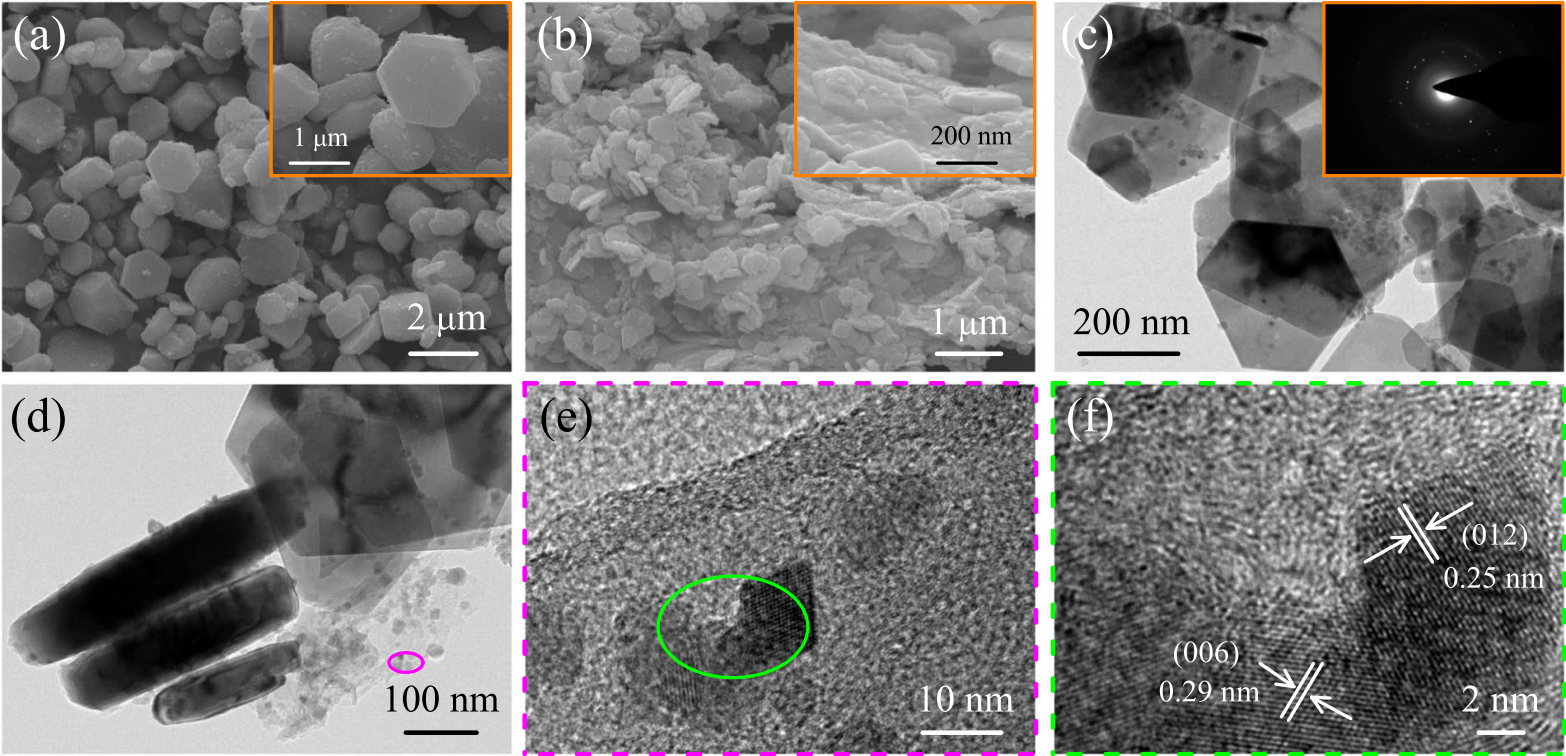
(**a**) SEM image of CuFeO_2_, inset shows the higher magnification. (**b**) SEM image of CuFeO_2_@rGO, inset shows the higher magnification. (**c**,**d**) TEM images of CuFeO_2_@rGO, inset of (**c**) shows the selected area electron diffraction (SAED) pattern. (**e**) High magnitude TEM image of CuFeO_2_@rGO. (**f**) HRTEM image of CuFeO_2_@rGO.

The morphology and microstructure of the as-prepared CuFeO_2_ and CuFeO_2_@rGO were characterized by scanning electron microscopy (SEM) and transmission electron microscopy (TEM). Figure 3a reveals the large hexagonal platelet characteristic of 1–2 *μ*m in diameter and 300–600 nm in thickness for bare CuFeO_2_. Figure 3b–d show that the hexagonal CuFeO_2_ crystals with the diameter of 200–400 nm and thickness of 40–60 nm are homogeneously anchored on the surface of graphene nanosheets for CuFeO_2_@rGO. The reduced size of CuFeO_2_ can be attributed to the functional groups of graphene nanosheets, which can not only restrict the size of CuFeO_2_ but also act as nucleation centers to facilitate the formation of crystals^32, 61^. The smaller size of CuFeO_2_ attached on graphene nanosheets is advantageous to facilitate the lithium ion diffusion and accommodation the large volume changes during cycling, resulting in better electrochemical performance for LIBs. In addition, the selected area electron diffraction (SAED) pattern (inset of Fig. 3c) shows a set of well-defined spots of the prepared CuFeO_2_ on rGO. A magnified TEM image (Fig. 3e) clearly shows that the primary CuFeO_2_ nanocrystals are formed on graphene, which further confirms the growth mechanism of CuFeO_2_. The high-resolution TEM (HRTEM) image reveals clear lattice fringe spacing of 0.25 nm and 0.29 nm (Fig. 3f), which can be readily indexed to the (012) and (006) planes of the delafossite CuFeO_2_ crystal, respectively.

**Figure 4:**
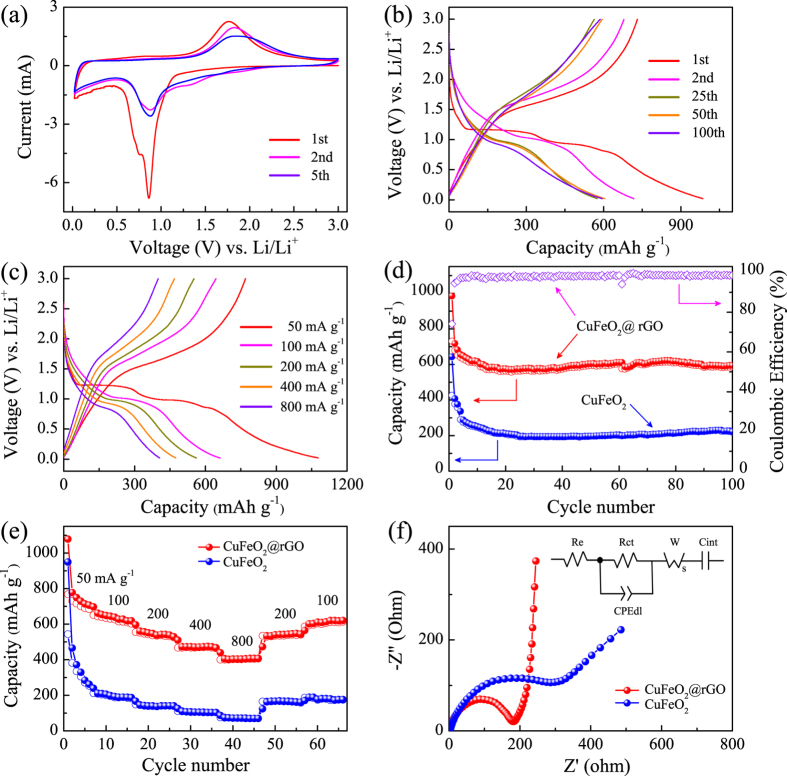
(**a**) CV curves of CuFeO_2_@rGO. (**b**) Charge/discharge voltage profiles of CuFeO_2_@rGO at 200 mA g^−1^. (**c**) Charge/discharge voltage profiles of CuFeO_2_@rGO at different rates. (**d**) Cycling performance of CuFeO_2_@rGO and CuFeO_2_ at 200 mA g^−1^ for 100 cycles. (**e**) Rate capabilities of CuFeO_2_@rGO and CuFeO_2_ at different current density. (**f**) Nyquist plots and equivalent circuit of CuFeO_2_ and CuFeO_2_@rGO.

As a demonstration, the as-prepared CuFeO_2_ composites were employed as anodes for LIBs. The cyclic voltammetry (CV) analysis was applied to obtain the electrochemical details at a scan rate of 0.5 mV s^−1^ within a voltage window of 0.02–3 V (vs. Li/Li^+^). Figure 4a shows the 1st, 2nd, and 5th CV curves of CuFeO_2_@rGO electrode. In the first cathodic process, two obvious peaks at about 0.86 and 0.75 V can be ascribed to the decomposition of electrolyte, the formation of solid electrolyte interface (SEI) layer on the electrode surface, as well as the irreversible reduction of CuFeO_2_ (CuFeO_2_ + 4Li^+^ + 4e^−^ → Cu + Fe + 2Li_2_O). In the reversed anodic process, the broad oxidation peak centered at 1.78 V represents the reversible oxidation of metallic Cu and Fe (Cu + 2Fe + 4Li_2_O ↔ Cu_2_O + Fe_2_O_3_ + 8Li^+^ + 8e^−^) and Li_2_O decomposition. In the subsequent cycles, the cathodic peak located at 0.87 V corresponds to the reduction of Cu_2_O, Fe_2_O_3_ and the formation of SEI film^31^. The corresponding CV curves of CuFeO_2_ are shown in Fig. S4. The only peak at about 0.73 V in the first cathodic process should be assigned to the reduction of CuFeO_2_ and the irreversible reaction related to the decomposition of the electrolyte^32^. The different in the first discharge cycle between CuFeO_2_ and CuFeO_2_@rGO may be attributed to the synergistic effects of graphene^62^. Figure 4b shows the charge/discharge cycling of CuFeO_2_@rGO electrode in the initial, second, twenty-fifth, fiftieth and hundredth cycles at the current density of 200 mA g^−1^. It can be seen clearly that the voltage drops sharply from the open-circuit voltage to about 1.2 V during the first discharge cycle, which is corresponding to the beginning the insertion of Li^+^ ions^63^. The discharge profile mainly consists of voltage plateau at about 1.2 V and 0.95 V, agreeing with the first CV curve. At about 0.8 V, the voltage starts dropping with a gentle sloping profile. The first charge cycle has no voltage plateau but a sloping profile that changes at about 1.5 V till about 2.3 V, before changing again. The second charge cycle is analogous to the first charge cycle, which indicates that similar electrochemical reactions are taking place in both cycles. The second discharge cycle has a very different profile in comparison with the first discharge cycle, indicating disparate electrochemical reactions. The voltage plateau originally seen at 1.2 V is no longer seen. The voltage drops slowly from 3 to 1 V, and then slopes downward till 0.02 V. Moreover, the initial discharge capacity of CuFeO_2_@rGO (985 mAh g^−1^) is remarkably higher than the theoretical capacity of CuFeO_2_ (708 mAh g^−1^), which have been found in other metal oxides^12, 13, 32, 47, 57^. The higher initial discharge capacity may be ascribed to structural destruction upon Li insertion and decomposition of the solvent in the electrolyte, subsequent formation of large area solid electrolyte interphase (SEI) layer and nano Cu and Fe in Li_2_O matrix. The initial charge capacity of CuFeO_2_@rGO is 730 mAh g^−1^, yielding a coulombic efficiency of 74%. The formation of SEI layer on the surface of active materials has been recognized as the primary cause for irreversible capacity loss, including graphene (Fig. S5)^3, 4, 48^. Moreover, the high irreversible capacity loss can also be attributed to the volume variations, some undecomposed Li_2_O phase, along with the irreversible reduction of active materials and electrolyte during the first discharge process^11, 64–66^. Notably, the curves are strongly overlapped for 25, 50 and 100 cycles, suggesting the good stability and reversibility of CuFeO_2_@rGO electrode.

The galvanostatic charge/discharge (GCD) profiles at various rate and the corresponding comparison are shown in Fig. 4c and Fig. S6. One can see that the CuFeO_2_@rGO electrode delivers discharge capacities of 1078, 561 and 406 mAh g^−1^ at 50, 200 and 800 mA g^−1^, respectively. In contrast, not only the initial capacity of bare CuFeO_2_ electrode is lower than the CuFeO_2_@rGO electrode, but there is also severe capacity fading in a reversible capacity of only 146 mAh g^−1^ at 200 mA g^−1^. It indicates that the introduction of graphene is beneficial for fast charge transfer and the electrode stable at high rate, leading to the enhancement of electrochemical capability. Fig. 4d shows the cycling performance of CuFeO_2_@rGO and bare CuFeO_2_ electrodes at the current density of 200 mA g^−1^. The CuFeO_2_@rGO electrode demonstrates excellent reversibility and cycling stability. A reversible capacity as high as 587 mAh g^−1^ is sustained after 100 cycles. It is worth noting that the capacity fading from the first to the tenth cycles can be ascribed to the complicated side-reactions and irreversible structure transformation^67, 68^. However, in the case of bare CuFeO_2_, the reversible capacities seriously decline to 222 mAh g^−1^, which can be ascribed to the large volume expansion and mechanical stress during lithiation/delithiation processes^31^. Moveover, the CuFeO_2_@rGO electrode exhibits a superior cycling capability and stability even at a high rate of 800 mA g^−1^ (400 mAh g^−1^ after 100 cycles) (Fig. S7). The significantly enhanced cycling performances are closely related to the sheet-on-sheet architecture of CuFeO_2_@rGO. Specifically, the small size of CuFeO_2_ along with the high surface area of graphene can provide more electrochemical reaction sites and suppress the aggregation of active materials to keep electrode structure stable^69^. Impressively, the CuFeO_2_@rGO electrode behaves robust rate capability (Fig. 4e). Upon cycling at various current densities of 50, 100, 200, 400, 800 mA g^−1^, the CuFeO_2_@rGO electrode exhibits average discharge capacities of 720, 630, 538, 474, 406 mAh g^−1^, respectively. Furthermore, the discharge capacity could maintain a discharge capacity of 613 mAh g^−1^ when the rate is returned to 100 mA g^−1^. In contrast, the CuFeO_2_ electrode shows inferior rate capability, achieving mere 70 mAh g^−1^ at 800 mA g^−1^ and poor recovery (178 mAh g^−1^ at 100 mA g^−1^).

The inspiring rate capability and cycling stability of CuFeO_2_@rGO electrode originate from the sheet-on-sheet structure. In CuFeO_2_ electrode, limited by the inherent poor conductivity and sluggish ion transport of disconnected micron-grade crystals, the lithium ion cannot effectively diffuse to the active materials through electrolyte. In contrast, the interconnected CuFeO_2_@rGO architecture with nanoscale CuFeO_2_ can shorten the Li^+^ diffusion pathway for fast electron/ionic transportation. In addition, The conductive graphene boosts the electrical conductivity and the sufficient contact between electrolyte and active materials, promoting charge transfer at the electrode/electrolyte interface. To further understand the electrode kinetics mechanism of CuFeO_2_@rGO and CuFeO_2_, electrochemical impedance spectra (EIS) were carried out on the fresh cells from the open circuit voltage. The Nyquist plots are shown in Fig. 4f, all spectrum consist of a depressed semicircle and a straight line. The semicircle is related to charge transfer resistance whereas the line corresponds to solid state diffusion resistance^64, 66^. The spectra were fitted to an equivalent circuit consisting of resistances (both electrolyte R_*e*_ and charge transfer R_*ct*_), a constant phase element (CPE), a Warburg impedance (W_*s*_) and an intercalation capacitance (C_*int*_). W_*s*_ is associated with the solid-state diffusion resistance^70, 71^. The values of the circuit elements shown in Table S1 (Supporting Information) confirm the easy lithiation kinetics of CuFeO_2_@rGO electrode. Moreover, the sheet-on-sheet configuration with high surface area can offer more electrochemical reaction sites, which are benefit for the lithiation/delithiation reaction of active materials. The configuration also provides more volume to prevent the aggregation of active materials, ensuing a stable electrode structure. All the aforementioned factors contribute the remarkable electrochemical lithium storage properties of the CuFeO_2_@rGO composites.

### The Cu/CuFe_2_O_4_@rGO composites

The conductive additive has shown great success for promoting the capacity and stability of delafossite-type CuFeO_2_ based anode. Such a strategy should be extended to spinel-type copper ferrite CuFe_2_O_4_ with higher theoretical capacity (895 mAh g^−1^). However, the active materials could be separated from the add-in graphene for the weak interaction during high-rate cycling, which can be ameliorated by the combination with metal nanocrystals^72, 73^. In particular, metallic copper with high electrical conductivity has been proven to be an efficiently additive for advanced energy storage. Therefore, the Cu/CuFe_2_O_4_@rGO electrode is expected to constructed though a one-step hydrothermal approach, as shown in Fig. 1b. In this reaction process, ethylene glycol medium can act not only as a solvent, but also as a reductant to induce the incorporation of metallic Cu. Moreover, as a cosolvent, ethylenediamine has a stronger chelating ability for the release of isolated iron ions, which influences the crystal growth and generates the CuFe_2_O_4_. The integration of metal copper and graphene with CuFe_2_O_4_ nanocrystals can prevent the exfoliation of active materials and accelerate the transportation of electrons/ion. Thus, the prospection of higher capacity and high-rate performance for copper ferrites based lithium storage can be achieved.

**Figure 5:**
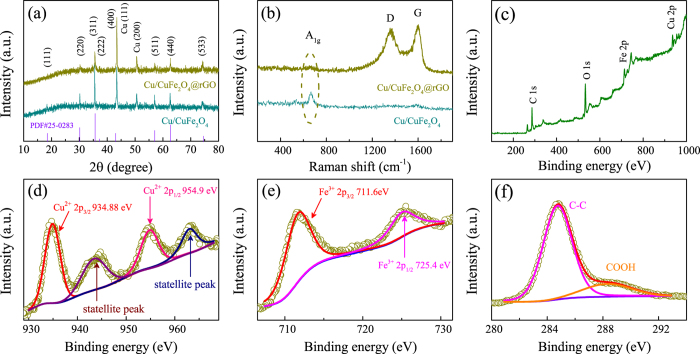
(**a**) XRD patterns and (**b**) Raman spectra of Cu/CuFe_2_O_4_ and Cu/CuFe_2_O_4_@rGO. (**c**) XPS survey spectrum of Cu/CuFe_2_O_4_@rGO. High-resolution XPS spectra of (**d**) Cu 2*p*, (**e**) Fe 2*p*, (**f**) C 1 *s* for Cu/CuFe_2_O_4_@rGO.

The crystallographic structure of the as-fabricated Cu/CuFe_2_O_4_@rGO hybrid was analyzed by XRD technique. As shown in Fig. 5a, the strong diffraction peaks at around 43° and 51° can be assigned as the (111) and (200) peak of Cu (PDF# 04-0836), respectively. The other diffraction peaks can be indexed as spinel CuFe_2_O_4_ (PDF# 25-0283), which confirms the good crystallinity of the products. Moreover, Cu/CuFe_2_O_4_ and Cu/CuFe_2_O_4_@rGO show the similar lattice parameter values of a = b = c = 0.8373 nm. Raman spectroscopy was performed to analyze the coating conditions of rGO layer (Figs 5b and S8). The band at 665 cm^−1^ corresponds to the A_1*g*_ vibration of CuFe_2_O_4_. Compared with GO (*I*_*D*_/*I*_*G*_ = 0.86), the increased D/G intensity ratio (*I*_*D*_/*I*_*G*_ = 0.94) in Cu/CuFe_2_O_4_@rGO suggests the reduction of graphene^57^. The presence of 2D band shows a substantial increase in the disorder degree of graphene with many layers^32, 50^. The specific surface area of Cu/CuFe_2_O_4_ composites show in Fig. S9. The BET value of Cu/CuFe_2_O_4_ is 11.96 m^2^ g^−1^, similar to the other metal oxides with analogous morphology^16, 23^. The high specific surface area of Cu/CuFe_2_O_4_@rGO (161.39 m^2^ g^−1^) is mainly the contribution of graphene, which is connected to large SEI formation, extending up to very high capacity. XPS measurements were conducted to further evaluate the composition of Cu/CuFe_2_O_4_@rGO composites. The survey XPS spectrum (Fig. 5c) clearly indicates the presence of Cu, Fe, O and C elements, consistent with the above XRD and Raman results. The high-resolution Cu 2p spectra (Fig. 5d) reveals Cu^2+^ 2p_3/2_ and Cu^2+^ 2p_1/2_ binding energy peaks at 934.8 eV and 954.9 eV, respectively^74^. The statellite peaks at 944 eV and 963 eV indicate the existence of metallic copper in Cu/CuFe_2_O_4_@rGO hybrid. From Fig. 5e, the peaks at 711.6 eV and 725.4 eV with an energy difference of 13.8 eV, are assigned to Fe^3+^ 2p_3/2_ and Fe^3+^ 2p_1/2_, respectively. Moreover, the strong C 1 s peak located at 284.5 eV is assigned to the graphitic carbon (C-C) of rGO whereas the weaker peak at 288.5 eV is related to the C in carboxyl (COOH) (Fig. 5f)^30^. The dramatic loss of oxygen-containing functional groups further indicates the deoxygenation process accompanying the reduction of GO, which is ascribed to the addition of reductive agent in the hydrothermal reaction.

**Figure 6:**
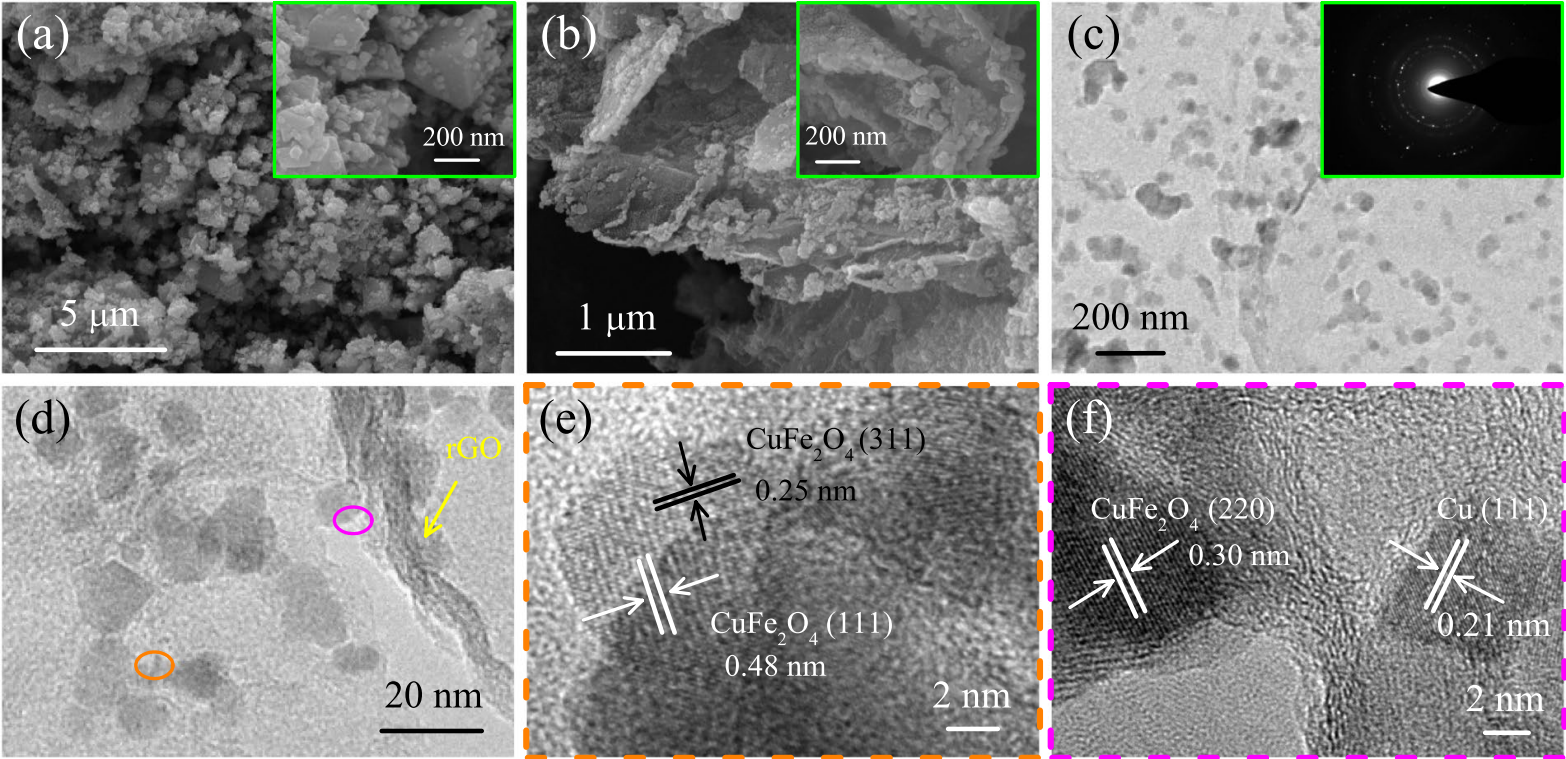
(**a**) SEM image of Cu/CuFe_2_O_4_, inset shows the higher magnification. (**b**) SEM image of Cu/CuFe_2_O_4_@rGO, inset shows the higher magnification. (**c**) TEM image of Cu/CuFe_2_O_4_@rGO, inset shows the selected area electron diffraction (SAED) pattern. (**d**) High magnitude TEM image of Cu/CuFe_2_O_4_@rGO. (**e**,**f**) HRTEM images of Cu/CuFe_2_O_4_@rGO.

The morphology and microstructure of Cu/CuFe_2_O_4_@rGO composites were examined by SEM and TEM. It can be seen from Fig. 6a and Fig. S10 that the as-prepared Cu/CuFe_2_O_4_ has a great irregular CuFe_2_O_4_ around large metallic copper crystals. The typical SEM images at different magnifications are shown in Fig. 6b and Fig. S11. It clearly reveals the uniform morphology over the whole surface of Cu/CuFe_2_O_4_@rGO, wherein graphene nanosheets have a mass of ultrafine nanoparticles evenly anchored on them. Notably, there is no large aggregations of CuFe_2_O_4_ and metallic copper or large vacancies in graphene nanosheets, exhibiting a better distribution and smaller size in comparison with Cu/CuFe_2_O_4_. The huge transformation of morphology between the two samples can be ascribed to the synergistic effects of graphene, EN and EG used in the hydrothermal reaction. Graphene can be decomposed by EN and reduced by EG, resulting in abundant active sites, which can control the crystal nucleation and growth of CuFe_2_O_4_ and Cu^75^. In addition, the chelates of EG and the large surface areas of graphene can prevent the agglomeration during the particle growth process^76^. The TEM images further confirm that graphene nanosheets are decorated by ultrafine nanoparticles with the diameter of 15–25 nm, which interconnected through rGO (Fig. 6c,d). The selected area electron diffraction (SAED, inset of Fig. 6c) shows the polycrystalline diffraction rings of Cu/CuFe_2_O_4_@rGO composites. The HRTEM images (Fig. 6e,f) recorded on two different parts further confirm the formation of Cu/CuFe_2_O_4_@rGO. The lattice fringes of 0.25 nm and 0.48 nm in Fig. 6e can be ascribed to the (311) and (111) plane of CuFe_2_O_4_. The interplanar spacing of 0.21 nm and 0.30 nm in Fig. 6f can be assigned to the (111) plane of Cu and (220) plane of CuFe_2_O_4_. The high surface area of Cu/CuFe_2_O_4_@rGO combined with uniform distribution of metal copper and ultrafine CuFe_2_O_4_ is helpful for fast ion access, stable crystalline structure and efficient electrolyte penetration. Moreover, the hybrid system can promote the electrochemical activities and prevent the separation of CuFe_2_O_4_ during cycling, which can result in high-rate lithium storage performance.

**Figure 7:**
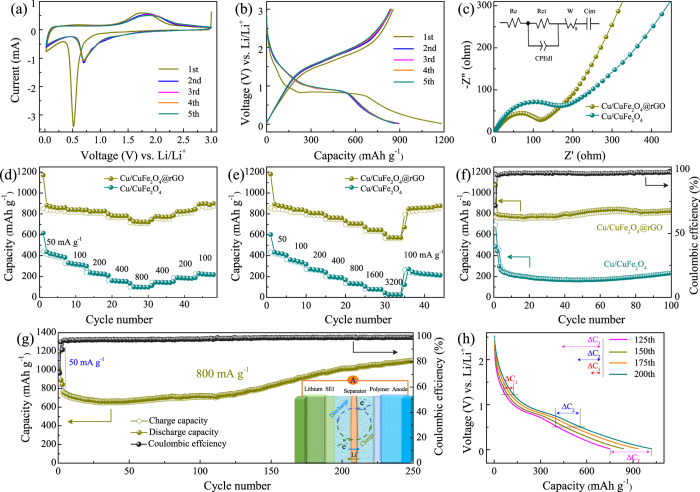
(**a**) CV curves and (**b**) charge/discharge voltage profiles of Cu/CuFe_2_O_4_@rGO at 50 mA g^−1^ for the initial five cycles. (**c**) Nyquist plots and equivalent circuit of Cu/CuFe_2_O_4_ and Cu/CuFe_2_O_4_@rGO. (**d**) Rate capabilities of Cu/CuFe_2_O_4_@rGO and Cu/CuFe_2_O_4_ at different current density. (**e**) Rate capabilities of Cu/CuFe_2_O_4_@rGO and Cu/CuFe_2_O_4_ at varying rate from 50 mA g^−1^ to 3200 mA g^−1^. (**f**) Cycling performance of Cu/CuFe_2_O_4_@rGO and Cu/CuFe_2_O_4_ at 200 mA g^−1^ for 100 cycles. (**g**) Cycling performance of Cu/CuFe_2_O_4_@rGO at 800 mA g^−1^ for 250 cycles, inset shows the schematic illustration of the half-cell structure during cycling. (**h**) Selected discharge voltage profiles of Cu/CuFe_2_O_4_@rGO at 800 mA g^−1^.

The CV profiles of as-prepared CuFe_2_O_4_ based electrodes are shown in Fig. 7a and Fig. S12. For Cu/CuFe2O4@rGO electrode, the cathodic sharp peak located at around 0.5 V in the first cycle corresponds to the irreversible reduction of CuFe_2_O_4_ (CuFe_2_O_4_ + 8Li^+^ + 8e^−^ → Cu + 2Fe + 4Li_2_O), as well as the growth of SEI layer^33^. Two broad overlapping anodic peaks positioned at about 1.74 and 1.85 V can be attributed to the reversible oxidation of metallic Cu and Fe (Cu + 2Fe + 4Li_2_O ↔ CuO + Fe_2_O_3_ + 8Li^+^ + 8e^−^), as well as the SEI decomposition^34^. In subsequent cycles, the cathodic/anodic peaks at around 0.7 V/1.85 V can be observed, corresponding to the improved kinetics as well as the lithiation/delithiation reactions of CuO/Cu and Fe_2_O_3_/Fe. Note that the voltammograms are superimposable perfectly after the first cycle, as compared with Cu/CuFe_2_O_4_ and CuFe_2_O_4_, suggesting better electrochemical reactivity and reversibility for Cu/CuFe_2_O_4_@rGO. The initial five voltage profiles of Cu/CuFe_2_O_4_@rGO electrode at 50 mA g^−1^ and the corresponding comparison are shown in Fig. 7b and Fig. S13. The voltage drops sharply from the open-circuit voltage to about 0.85 V during the first discharge cycle for all electrodes. There is a voltage plateau at about 0.85 V, followed by a sloping profile till just above 0.02 V. The second discharge cycle had a different profile. The voltage dropped initially to about 1 V and has a small voltage plateau at around 0.95 V. At about 0.85 V, the voltage decreased steeply to 0.02 V. The charge cycles are similar and all has a sloping profile that changes at about 1.5 V till 2.3 V. Moreover, the Cu/CuFe_2_O_4_@rGO delivers an initial discharge and charge capacity of 1169 mAh g^−1^ and 855 mAh g^−1^ with a first coulombic efficiency of 73.1%, much higher than those for Cu/CuFe_2_O_4_ and CuFe_2_O_4_. The enhanced capacity is attributed to the contribution of graphene and metallic copper along with smaller crystal size, which can increase the utilization of active materials. The large irreversible capacity loss is likely ascribed to the consumption of Li^+^ to form an irreversible SEI layer and the reduction of CuFe_2_O_4_. Noticeably, smaller voltage hysteresis for Cu/CuFe_2_O_4_@rGO electrode manifests its better electrochemical stability. EIS were carried out on the fresh cells from the open circuit voltage to understand the kinetics of lithiation and delithiation and resistance to charge transfer. The typical Nyquist plots in which semicircles and Warburg line are present are shown in Fig. 7c along with the respective equivalent electrical circuits (the values of the circuit elements are shown in Table S2). The overall low impedance values imply the better reaction kinetics of Cu/CuFe_2_O_4_@rGO electrode^64^. The availability of Cu/CuFe_2_O_4_@rGO with Cu and CuFe_2_O_4_ nanoparticles can effectively reduce the ion transport dimensions and enlarge the contact surface of electrode-electrolyte, resulting in improved electrochemical performance.

As shown in Fig. 7d, the Cu/CuFe_2_O_4_@rGO electrode shows an enhanced rate capability, with the average discharge capacity of 863 mAh g^−1^ and 723 mAh g^−1^ at rates of 50 mA g^−1^ and 800 mA g^−1^. Moreover, the capacity can recover to the initial value as long as the rate reverses back to low current density, highlighting the cycling durability. For comparison, the Cu/CuFe_2_O_4_ electrode delivers mere 100 mAh g^−1^ at 800 mA g^−1^ and exhibits poor recovery. To further evaluate the ultrafast electrochemical ability of Cu/CuFe_2_O_4_@rGO electrode, high-rate testing was conducted at even higher current densities from 50 mA g^−1^ to 3200 mA g^−1^ (Fig. 7e). Noticeably, the Cu/CuFe_2_O_4_@rGO electrode features a high-rate reversible capability as well as stability and the capacity maintains above 560 mAh g^−1^ at 3200 mA g^−1^. For comparison, the Cu/CuFe_2_O_4_ electrode demonstrates much poor rate capability with negligible discharge capacity of 27 mAh g^−1^ at 3200 mA g^−1^. In particular, the Cu/CuFe_2_O_4_@rGO electrode can quickly recover to its original capacity or even higher (861.1 mAh g^−1^ in the 40th cycle) when the rate abruptly switched to 100 mA g^−1^, indicating the promising application for advanced energy storage. The significantly boosted rate capacity of Cu/CuFe_2_O_4_@rGO electrode is mainly induced by the synergistic effect of graphene sheets and small crystal. The conductive rGO and metallic copper enhance the electronic conductivity and shorten electronic/ionic transport length, resulting in better lithiation/delithiation reaction kinetics. Moreover, the large surface area of Cu/CuFe_2_O_4_@rGO provides more surface to the electrolyte and activation sites for electrochemical reactions. In addition, the large surface area can effectively buffer the large volume change during lithium reactions, ensuring a superior high-rate performance.

In addition to ultrahigh rate capability, the Cu/CuFe_2_O_4_@rGO electrode also possesses boosted cycling performance. As shown in Fig. 7f, the Cu/CuFe_2_O_4_@rGO electrode delivers a reversible capacity of 835.2 mAh g^−1^ after 100 cycles at 200 mA g^−1^, which is much higher than that for Cu/CuFe_2_O_4_ (235 mAh g^−1^ after 100 cycles). It suggests the extraordinary cycling stability for the highly reversible Li^+^ insertion/extraction kinetics. Furthermore, the long-term high-rate cycling performance of Cu/CuFe_2_O_4_@rGO was evaluated at 800 mA g^−1^ for 250 cycles after being activated for 3 cycles at 50 mA g^−1^ (Fig. 7g). An ultrahigh discharge capacity of 1102 mAh g^−1^ is yielded even after 250 cycles, indicating the robust cyclability even under the long-term and fast discharge/charge cycling. Interestingly, there is a capacity increasing stage after a slow capacity decay, which has been reported for most transition metal oxides^62, 77^. Such an activation behavior for LIBs originates from the gradually emerging interfacial storage contribution, which can be attributed to the faradaic contribution of pseudo-capacitance and non-faradaic contribution of double-layer capacitance^78^. In addition, the reversible growth of a polymeric gel-like film around the active materials from electrolyte degradation also can lead to the gradually increased capacity, as shown in the inset of Fig. 7g. The comparison in electrochemical properties between Cu/CuFe_2_O_4_@rGo and other related works has been summarized in Table S3. In order to reveal the capacity changes with cycling, some selected discharge profiles of Cu/CuFe_2_O_4_@rGO at 800 mA g^−1^ are shown in Fig. 7h. According to the CV test at a scan rate of 0.5 mV s^−1^ after the frst lithiation/delithiation cycle (Fig. 7a), the discharge processes can be roughly divided into three voltage ranges of 1.2–3.0 V (ΔC_1_), 0.5–1.2 V (ΔC_2_–ΔC_1_), 0.02–0.5 V (ΔC_3_–ΔC_2_), respectively. Those three stages are corresponding to the formation of gel-like polymer layer at low potentials, the reduction reaction of CuO and Fe_2_O_3_, the formation of gel-like polymer layer at high potentials, respectively. Obviously, the capacity increment at high potentials (ΔC_1_) is almost negligible. The whole capacity increment (ΔC_3_) is mainly dominated by the increasing capacity of the gel-like polymer layer formation at low potentials and the activation CuO and Fe_2_O_3_ reduction. The increasing capacity by the activation of CuO and Fe_2_O_3_ should be associated with the dispersion of metallic Cu nanoparticles in rGO matrix^54^. The presence of Cu nanoparticles with high surface activity can enhance the reversible electrochemical reaction of Li_2_O and the reversibility of Fe back to Fe_2_O_3_ during the charge process. In addition, the addition of copper increases extra reversibly convert of Cu_2_O during discharge cycling, making the capacity increment of Cu/CuFe_2_O_4_@rGO electrode with cycling^73^. Moreover, the capacity increment at low potentials may benefit from the enhanced gel-like polymer growth and the electrolyte decomposition. The formation of CuO and Fe_2_O_3_ nanoparticles upon cycling increases the specific surface area of the electrode and enhances the electrolyte decomposition, which could improve the capacity from the gel-like polymer growth^12^.

Generally, the increased electrochemical performances of Cu/CuFe_2_O_4_@rGO electrode can be ascribed to the synergistic effects among metallic Cu, CuFe_2_O_4_ particles and graphene nanosheets, originated from the specific electrode configuration. Firstly, the incorporated reduced graphene can offer nucleation sites for smaller crystal growth without any aggregation. The reduced size ensures the sufficient activation of active materials during reactions, resulting in the enhancement of capacity. Secondly, the highly uniform CuFe_2_O_4_ contacted with rGO offers interconnected ion diffusion pathways and adequate electrode/electrolyte interfacial area. It can facilitate lithium insertion/extraction to obtain superior rate performance. Thirdly, the highly conductive copper nanoparticles anchored on graphene nanosheets provide more electrochemical reaction sites and accommodate the volume change during cycling, which can lead to better cycling stability. The aforementioned advantages result in the significantly improved electrochemical performance of Cu/CuFe_2_O_4_@rGO, showing its potential application for advanced lithium storage.

## Conclusion

In summary, the copper ferrites@rGO anode materials for advanced lithium storage have been successfully prepared by a facile hydrothermal approach followed by a calcination process. The well-defined copper ferrites nanocrystals are uniformly capped with curved graphene. The synthetic effects of all components result in the enhancement of lithium storage performance through accelerating the electron/ion transfer and increasing the structural and interfacial stability. The CuFeO_2_@rGO electrode yields a high rate capability than bare CuFeO_2_. In particular, the facile fabricated Cu/CuFe_2_O_4_@rGO electrode can deliver a high reversible capacity of 1102 mAh g^−1^ after 250 cycles at a high current density of 800 mA g^−1^ and a remarkable rate capability among 50 to 3200 mA g^−1^. The resulting improvement of electron kinetics and appropriate spaces to alleviate the volume change is mainly responsible for the extraordinary performance of Cu/CuFe_2_O_4_@rGO. Such outstanding electrochemical performances make the copper ferrites based anode materials promising for the stationary energy storage systems.

## Methods

### Fabrication of CuFeO_2_@rGO

Graphene oxide (GO) was prepared using a modified Hummers method^79^. The CuFeO_2_ nanosheets and reduced graphene composites were fabricated by a facile hydrothermal approach and the subsequent calcination. In a typical procedure, 50 mg graphene nanosheets was first dissolved in 25 mL alcohol by mild sonication to form a uniform suspension. Subsequently, 1 mmol Fe(NO_3_)_3_ · 9H_2_O and Cu(NO_3_)_2_ · 2.5H_2_O were dissolved in 40 mL alcohol under constant magnetic stirring for 2 h in a separate flask to achieve a clear and homogeneous solution. Then the two solutions were mixed and the pH of the mixture was adjusted to 12 by 6 M NaOH under stirring. Thereafter, the mixture was transferred to a 100 mL Teflon-lined stainless steel autoclave and was hydrothermally treated at 180 °C for 12 h. After cooling to room temperature, the black precipitate was collected by centrifugation at 9000 rpm for 5 min, washed with deionized water and absolute alcohol alternately, and recollected by centrifugation several times. The final product was dried in vacuum at 60 °C for 12 h and calcined in a tube furnace at 400 °C for 2 h under a N_2_ atmosphere. For comparison, the pure CuFeO_2_ crystals were synthesized under the same conditions, but in the absence of graphene nanosheets.

### Fabrication of Cu/CuFe_2_O_4_@rGO

Briefly, 25 mL of graphene alcohol dispersion (2.5 mg mL^−1^) was mixed with 40 mL of 1 mmol Fe(NO_3_)_3_ · 9H_2_O and Cu(NO_3_)_2_ · 2.5H_2_O alcohol solution under vigorous magnetic stirring at room temperature. Then, the pH of the mixture was adjusted to 12 by 6 M NaOH and 5 mL of ethylene glycol (EG) and 5 mL of anhydrous ethylenediamine (EN) were added separately to the solution and stirred vigorously for 30 min. The solution was subsequently transferred into a 100 mL autoclave and maintained at 180 °C for 12 h. The resulting product was centrifuged, washed with deionized water and absolute alcohol several times and dried in vacuum at 60 °C for 12 h. The solid product was treated in the tube furnace at 400 °C for 2 h under a N_2_ atmosphere. For comparison, bare Cu/CuFeO_2_ was obtained by the similar procedures except for the absence of GO.

### Characterization methods

The phase purity and crystal structure were characterized by X-ray diffraction (XRD) using a Bruker D8 diffractometer with Cu-Ka radiation. The morphology and microstructure were investigated by scanning electron microscopy (SEM, PHILIPS XL30TMP) and transmission electron microscopy (TEM, FEI Tecnai G20). Raman spectra were recorded using a HORIBA Jobin Yvon Raman spectrometer with the excitation laser of 632.8 nm at room temperature. X-ray photoelectron spectroscopy (XPS) was conducted on a RBD upgraded PHI-5000C ESCA system (Perkin-Elmer) with Mg-K*α* radiation (*hν* = 1253.6 eV).

### Electrochemical measurements

The electrochemical measurements were recorded using coin-type 2032 cells. Working electrodes were prepared by pasting homogeneous slurries consisting of the active material (70 wt%), acetylene black (20 wt%), and polyvinylidene fluoride binder (10 wt%) dissolved in N-methyl-2-pyrrolidone onto pure Cu foil, followed by vacuum dried at 100 °C for 12 h. The coated Cu foil was punched into disks and used as the working electrodes. The cells were assembled using lithium metal as the counter/reference electrode, celgard 2400 polypropylene film as the separator in an Ar-filled glovebox (O_2_ and H_2_O contents <1 ppm). The electrolyte was 1 M LiPF6 dissolved in a mixture of ethylene carbonate, dimethyl carbonate, and diethyl carbonate (1:1:1, in vol%). The electrochemical performances of the cells were evaluated by galvanostatic charge/discharge (GCD) on a Land CT 2001A battery tester within a voltage range of 0.02–3 V (vs. Li/Li^+^). Cyclic voltammetry (CV) was conducted on a CHI-660D electrochemical workstation with a scan rate of 0.5 mV s^−1^ within a voltage window of 0.02C3 V (vs. Li/Li^+^). Electrochemical impedance spectroscopy (EIS) was performed within a frequency range of 100 kHz to 0.01 Hz by applying a sine wave with amplitude of 5 mV.

## Electronic supplementary material

Supporting Information
