# Supplementary material for: Copper ferrites@reduced graphene oxide anode materials for advanced lithium storage applications
Source: Sci Rep. 2017 Aug 21;7:8903. doi: 10.1038/s41598-017-09214-0 (PMC5566221; doi:10.1038/s41598-017-09214-0)
Supplement: Supplementary file 1 — Supporting Information [file 41598_2017_9214_MOESM1_ESM.pdf]

Supporting Information

Copper ferrites@reduced graphene oxide anode materials for advanced lithium storage applications

Junyong Wang, Qinglin Deng, Mengjiao Li, Kai Jiang, Jinzhong Zhang, Zhigao Hu*, **Junhao Chu**

Key Laboratory of Polar Materials and Devices (MOE) and Technical Center for Multifunctional Magneto-Optical Spectroscopy (Shanghai), Department of Electronic Engineering, East China Normal University, Shanghai 200241, China

*Corresponding author. *E-mail address:* zghu@ee.ecnu.edu.cn (Dated: Sunday 2nd July, 2017)

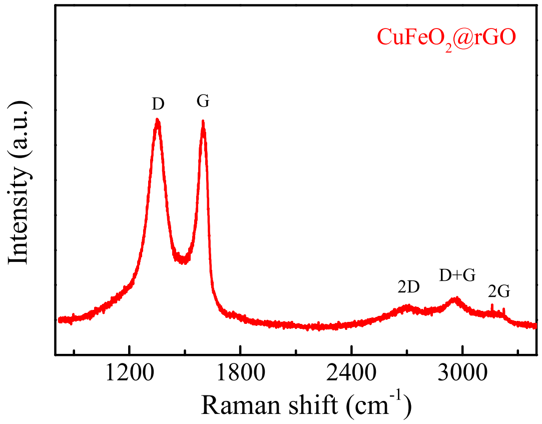

Fig. S 1: The Raman spectrum of $CuFeO_2$@rGO.

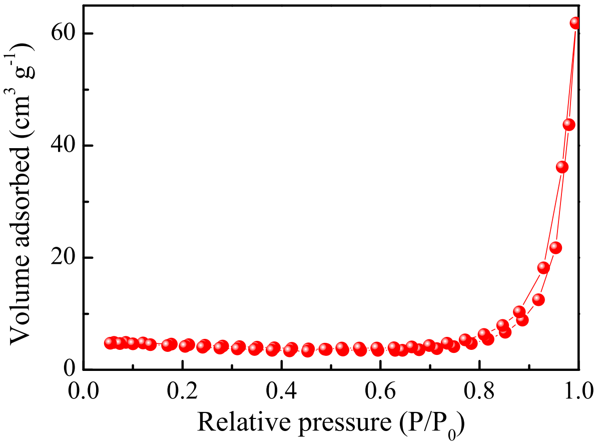

Fig. S 2: Nitrogen adsorption/desorption isotherms of $CuFeO_2$@rGO.

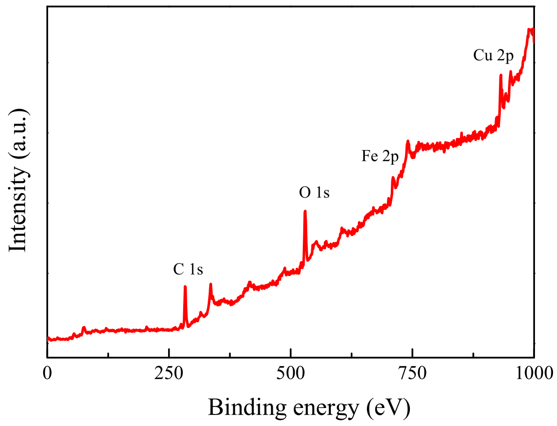

Fig. S 3: The full XPS spectrum of $CuFeO_2$@rGO.

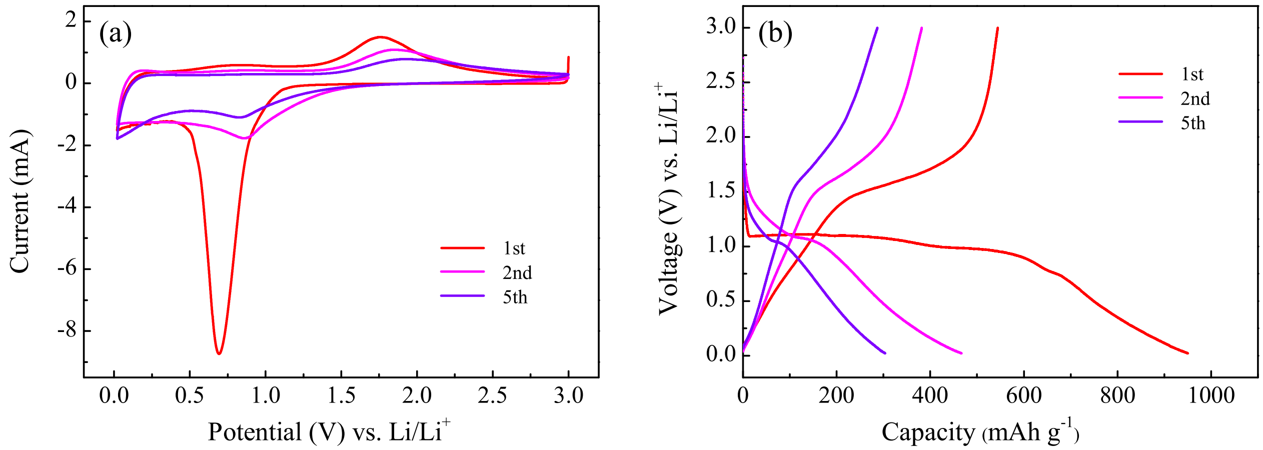

Fig. S 4: (a) Representative CV curves and (b) charge/discharge voltage profiles of $CuFeO_2$ at 50 mA g^{-1}.

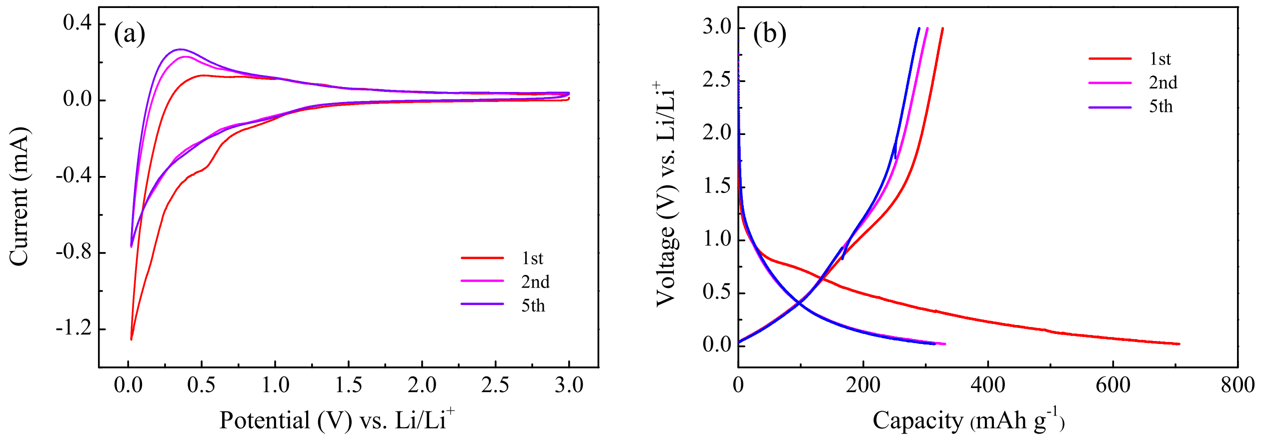

Fig. S 5: (a) Representative CV curves and (b) charge/discharge voltage profiles of graphene at 50 mA g^{-1}.

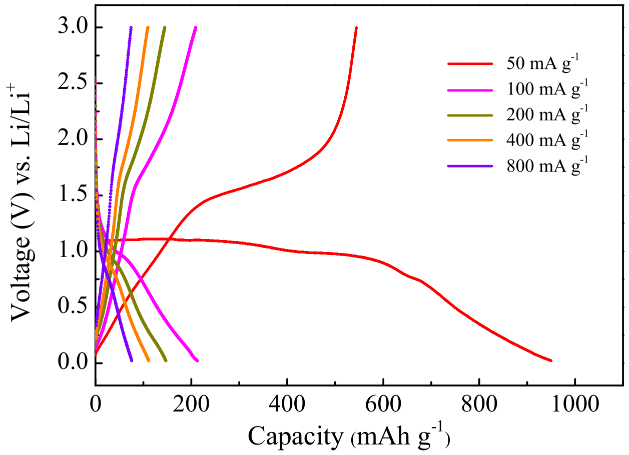

Fig. S 6: Charge/discharge voltage profiles of $CuFeO_2$@rGO under different current density.

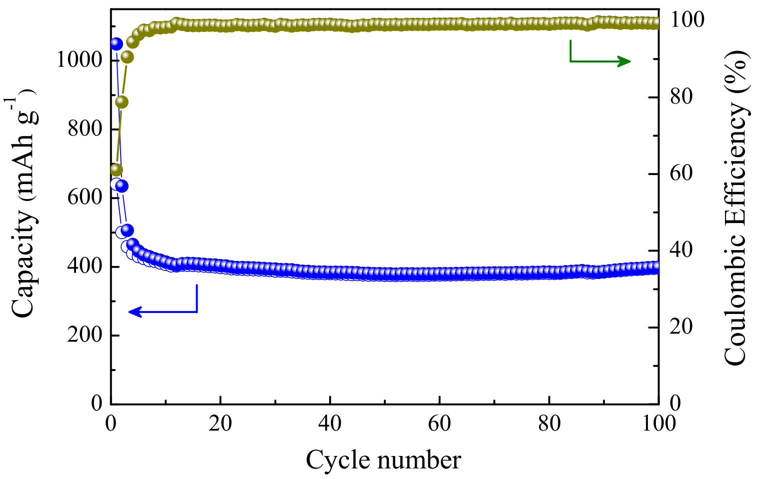

Fig. S 7: Cycling performance of $CuFeO_2$@rGO at 800 mA h^{-1} for 100 cycles.

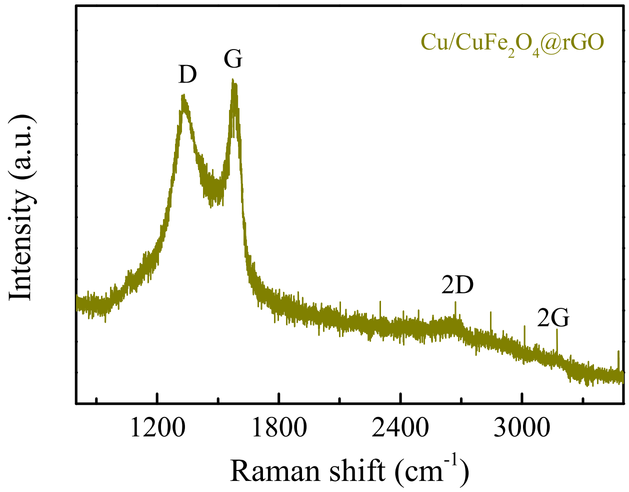

Fig. S 8: The Raman spectrum of Cu/$CuFe_2O_4$@rGO.

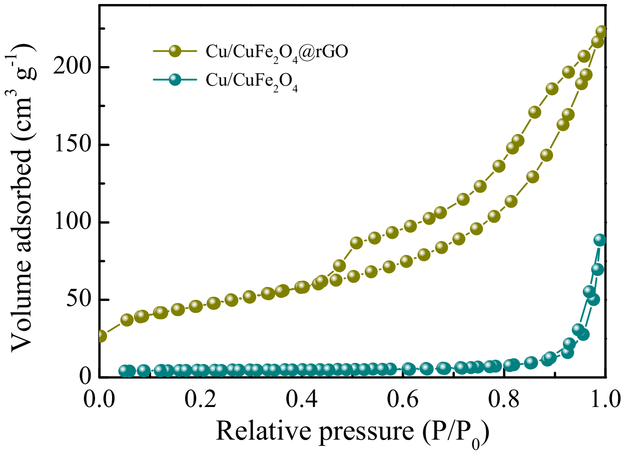

Fig. S 9: Nitrogen adsorption/desorption isotherms of Cu/$CuFe_2O_4$@rGO and Cu/$CuFe_2O_4$.

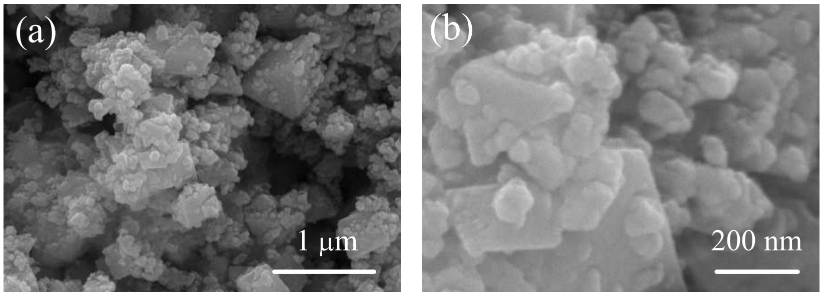

Fig. S 10: SEM images of $Cu/CuFe_2O_4$ at different magnifications.

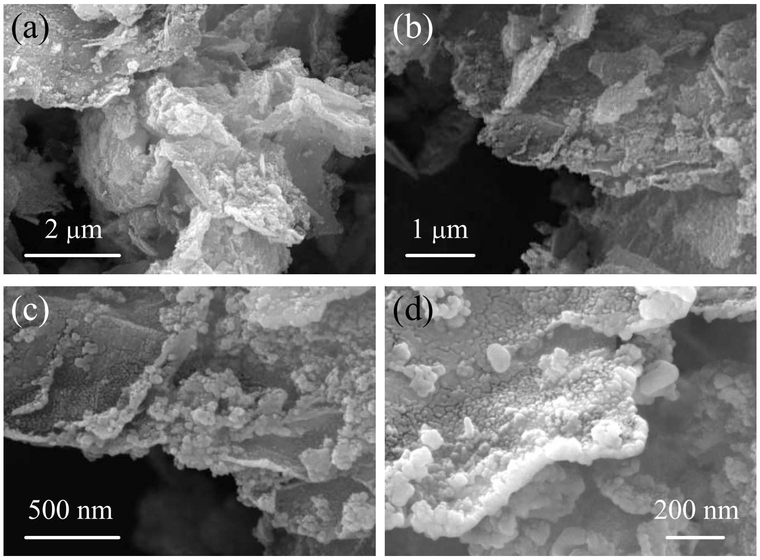

Fig. S 11: SEM images of $Cu/CuFe_2O_4$@rGO at different magnifications.

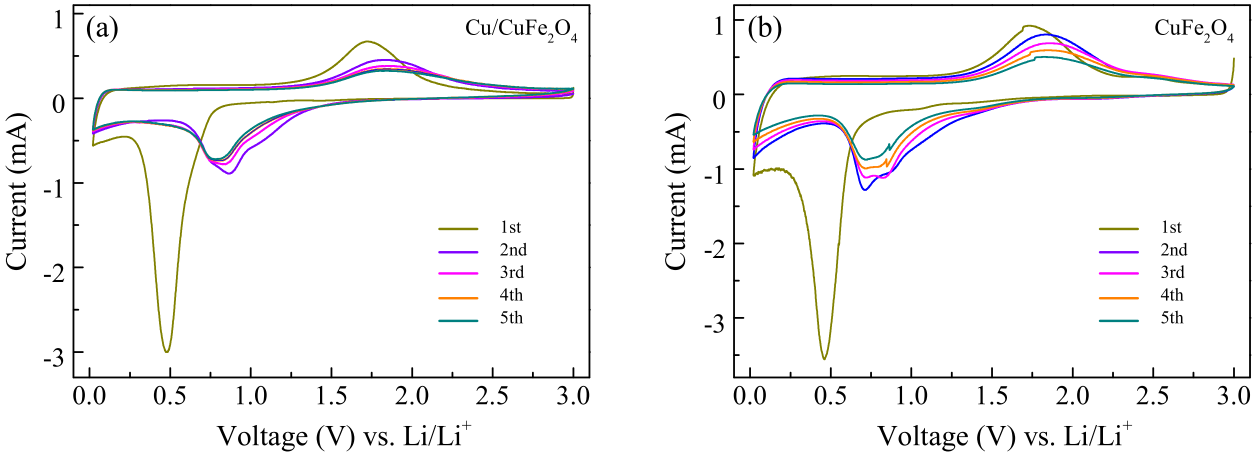

Fig. S 12: CV curves of (a) $Cu/CuFe_2O_4$ and (b) $CuFe_2O_4$ for the initial five cycles.

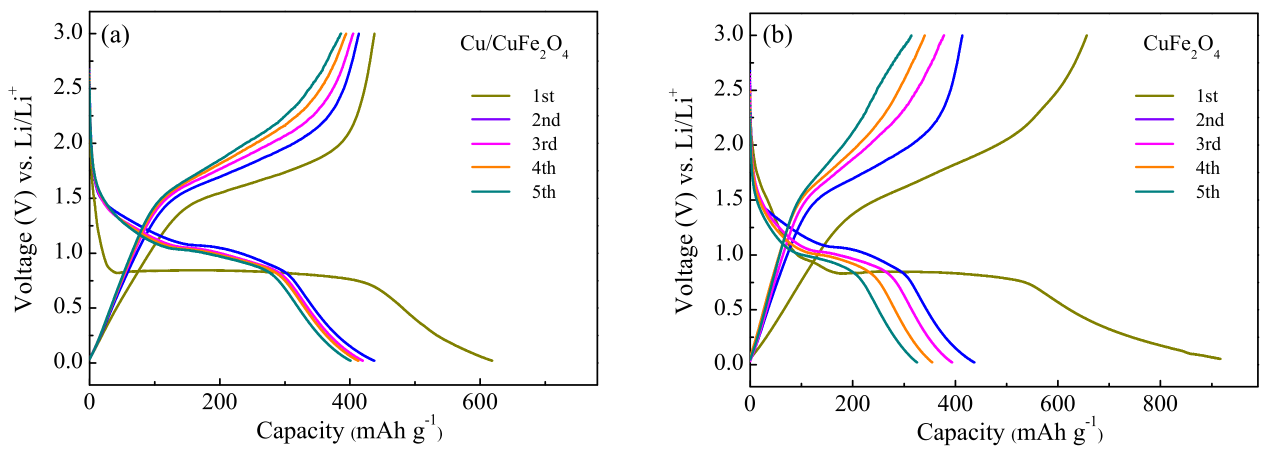

Fig. S 13: Charge/discharge voltage profiles of (a) $Cu/CuFe_2O_4$ and (b) $CuFe_2O_4$ at 50 mA g^{-1} for the initial five cycles.

Table. S1 Values of equivalent circuit elements obtained by EIS

Battery condition	R_e (Ω)	R_{ct} (Ω)	CPE_{dl} (μF)
$CuFeO_2$	1.939	248.8	14.29
$CuFeO_2$@rGO	1.971	169.9	8.25

Table. S2 Values of equivalent circuit elements obtained by EIS

Battery condition	R_e (Ω)	R_{ct} (Ω)	CPE_{dl} (μF)
$Cu/CuFe_2O_4$	3.071	147.3	60.17
$Cu/CuFe_2O_4$@rGO	1.948	115.5	21.05

Table. S3 Comparisons of the LIBs performance of $Cu/CuFe_2O_4$@rGO composites and recently reported metal oxides-based materials.

Sample	Current density ($mA\ g^{-1}$)	Reversible capacity ($mAh\ g^{-1}$)	Cycle number (Times)	References
$Cu/CuFe_2O_4$@rGO composites	800	1102	250	This work
3D graphene /Fe_2O_3 aerogel	200	1129	130	[S1]
Sandwich Structured Fe_2O_3 /Graphene Hybrids	200	1100	350	[S2]
Hollow Fe_2O_3 /N-Doped Graphene Aerogels	1000	729	300	[S3]
Free-standing CuO /rGO hybrid	67	736	50	[S4]
Core-shell structured Cu_2O@GO composite	100	458	50	[S5]
Hierarchical CNT/ Co_3O_4 microtubes	1000	784	200	[S6]
$CoFe_2O_4$ quantum dots /N-Doped Graphene	100	1223	90	[S7]
$ZnFe_2O_4$ nanoparticles @double graphene networks	500	706	1000	[S8]
$MnCo_2O_4$/graphene nanocomposites	200	865	50	[S9]
$CuCo_2O_4$ nanoparticles /rGO	1000	570	350	[S10]
Carbon-coated $CuCo_2O_4$ concave polyhedrons	1000	740	50	[S11]
$MnFe_2O_4$-rGO nanocomposite	1000	581	200	[S12]
Yolk-Shell MnO@$ZnMn_2O_4$ /N-C Nanorods	50	803	100	[S13]
$CuFe_2O_4$-Fe_2O_3 porous nanosheets	500	910	200	[S14]
Cu-$CuFe_2O_4$ -graphene composites	1000	672	200	[S15]

[S1] Jiang, T. C. *et al.* Porous Fe_2O_3 Nanoframeworks Encapsulated within Three-Dimensional Graphene as HighPerformance Flexible Anode for Lithium-Ion Battery. *ACS Nano* **11**, 5140-5147 (2017).

[S2] Qi, X. *et al.* Highly Efficient High-Pressure Homogenization Approach for Scalable Production of High-Quality Graphene Sheets and Sandwich Structured α-Fe_2O_3/Graphene Hybrids for High-Performance Lithium-Ion Batteries. *ACS Appl. Mater. Interfaces* **9**, 11025-11034 (2017).

[S3] Liu, L. *et al.* Seaweed-Derived Route to Fe_2O_3 Hollow Nanoparticles/N-Doped Graphene Aerogels with High Lithium Ion Storage Performance. *ACS Appl. Mater. Interfaces* **8**, 7047-7053 (2016).

[S4] Liu, Y. *et al.* Flexible CuO Nanosheets/Reduced-Graphene Oxide Composite Paper: Binder-Free Anode for High-Performance Lithium-Ion Batteries. *ACS Appl. Mater. Interfaces* **5**, 9850-9855 (2013).

[S5] Xu, Y. T. *et al.* Graphene oxide nano-sheets wrapped Cu_2O microspheres as improved performance anode materials for lithium ion batteries. *Nano Energy* **11**, 38-47 (2015).

[S6] Chen, Y. M., Yu, L. & Lou, X. W. Hierarchical Tubular Structures Composed of Co_3O_4 Hollow Nanoparticles and Carbon Nanotubes for Lithium Storage. *Angew. Chem. Int. Ed.* **5**, 5990-5993 (2016).

[S7] Yao, L. B. *et al.* Facile synthesis of $CoFe_2O_4$ quantum dots/N-doped graphene composite with enhanced lithium-storage performance. *J. Alloys and Compd.* **693**, 929-935 (2017).

[S8] Zhang, L. H. *et al.* Ultra-small and highly crystallized $ZnFe_2O_4$ nanoparticles within double graphene networks for super-long life lithium-ion batteries. *J. Mater. Chem. A* **5**, 11188-11196 (2017).

[S9] Chen, C. *et al.* Fabrication of cubic spinel $MnCo_2O_4$ nanoparticles embedded in graphene sheets with their improved lithium-ion and sodium-ion storage properties. *J. Power Sources* **326**, 252-263 (2016).

[S10] Kang, W. P. *et al.* Porous $CuCo_2O_4$ nanocubes wrapped by reduced graphene oxide as high-performance lithium-ion battery anodes. *Nanoscale* **6**, 6551-6556 (2014).

[S11] Ma, J. J. *et al.* Porous carbon-coated $CuCo_2O_4$ concave polyhedrons derived from metal-

organic frameworks as anodes for lithium-ion batteries. *J. Mater. Chem. A* **3**, 12038-12043 (2015).

[S12] Tang, H. *et al.* One-pot low-temperature synthesis of a $MnFe_2O_4$-graphene composite for lithium ion battery applications. *RSC Adv.* **4**, 28421-28425 (2014).

[S13] Zhong, M. *et al.* Yolk-Shell MnO@$ZnMn_2O_4$/N-C Nanorods Derived from α-MnO_2/ZIF-8 as Anode Materials for Lithium Ion Batteries. *Small* **12**, 5564-5571 (2016).

[S14] Ma, F. X. *et al.* Synthesis of self-stacked $CuFe_2O_4$-Fe_2O_3 porous nanosheets as a high performance Li-ion battery anode. *J. Mater. Chem. A* **2**, 19330-19337 (2014).

[S15] Dong, Y. C. *et al.* One-pot scalable synthesis of Cu-$CuFe_2O_4$/graphene composites as anode materials for lithium-ion batteries with enhanced lithium storage properties. *J. Mater. Chem. A* **2**, 13892-13897 (2014).
